# Editorial Comment on A rare case of long‐term survival from metachronous bilateral adrenal metastasis of lung adenocarcinoma after combined surgical removal and immunochemotherapy

**DOI:** 10.1002/iju5.12529

**Published:** 2022-08-26

**Authors:** Manabu Kato

**Affiliations:** ^1^ Department of Urology Aichi Cancer Center Hospital Nagoya Japan

In clinical practice, urologists encounter adrenal gland metastasis from other malignancies occasionally. Most cases are presented with unilateral metastatic lesions in the adrenal gland, although limited cases are demonstrated with bilateral adrenal metastasis. Usually, unilateral resection of the adrenal gland with metastatic lesion could be indicated in case the patient only has unilateral adrenal gland metastasis and clinically permitted general condition and acceptable prognosis due to adrenal function preservation even after unilateral adrenal surgical removal. Based on experience, patients with lung or gastric cancer are frequently consulted by the urologic department for adrenal metastasis treatment. Recently, both cancers have been attempted to be treated with immunotherapy and combined immunochemotherapy using a molecular target drug.^1^, ^2^ The national cancer comprehensive network guideline for nonsmall cell lung cancer has defined to use immunotherapy or immunochemotherapy drugs due to exhaustive gene expression analysis. For instance, descripted genetic abnormalities include sensitizing EGFR mutation, ALK rearrangement, ROS1 rearrangement, BRAF V600E mutation, NTRK gene fusion, and more than certain PD‐L1 expression percentage. Each genetic abnormality corresponds to one to six recommended treatment regimens orderly.^3^ Therefore, lung cancer could be positioned as a systematized disease regarding the standardization of genetic examination for personalized medicine treatment decision. From the perspective of clinical sample acquisition for genetic testing, adrenal metastasectomy is worthwhile for respiratory and oncology physicians who are mainly responsible for the patients. Besides lung cancer, other cancers have also started to be treated using the same methods.

In this case study, the patient underwent combined systemic lung cancer treatment and laparoscopic right adrenalectomy. Subsequent laparoscopic left adrenalectomy and systemic treatment led to the 4‐year progression‐free survival.^4^

The case study reminds urologists to have courage to conduct resection of both sides of the adrenal glands to achieve a cancer‐free condition with permanent adrenal hormone replacement after screening the patient's background, general conditions, and drug compliance.

## Conflict of interest

The authors declare no conflict of interest.
